# Influence of Composite Edible Coating of Pectin, Glycerol, and Oregano Essential Oil on Postharvest Deterioration of Mango Fruit

**DOI:** 10.1002/fsn3.4545

**Published:** 2024-11-21

**Authors:** Martha Sanchez‐Tamayo, José Luis Plaza‐Dorado, Claudia Ochoa‐Martínez

**Affiliations:** ^1^ Faculty of Agronomic Engineering University of Tolima Ibagué Colombia; ^2^ School of Food Engineering University of Valle Cali Colombia

**Keywords:** anthracnose control, mango coating, mixture design, optimal composition, postharvest deterioration

## Abstract

To control mango postharvest deterioration, an edible coating based on pectin, glycerol, and oregano essential oil (OEO) was developed using a three‐step process involving optimization, validation, and comparison with a commercial fungicide. An extreme vertices mixture design approach was initially used to optimize the pectin, glycerol, and OEO concentrations in the coating formulation. We evaluated the impact of the coating components on the respiration rate, quality parameters, and anthracnose disease of mangoes. The optimal coating composition was 3.91% (w/v) pectin, 0.57% (w/v) glycerol, and 0.52% (w/v) OEO, with a desirability value of 0.8998. Model validation demonstrated that the measured values for all parameters fit the prediction interval of 95%, and the relative error varied between 0.88% and 12.28%. Validation experiments of the optimal coating compared to untreated mango and mango treated with a commercial fungicide showed that the optimal coating delayed ripening and effectively controlled the incidence of anthracnose for 18 days at 14°C and 85% relative humidity.

## Graphical abstract

This study develops a pectin, glycerol, and oregano essential oil‐based coating for mango preservation, considering the limited options of postharvest treatments in producing countries and the growing demand for natural alternatives. This innovative method resulted in a coating that effectively delayed mango ripening and controlled anthracnose disease, presenting a viable alternative for mango preservation.

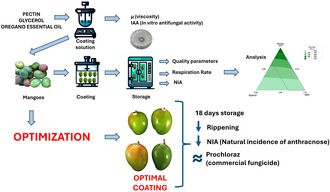

## 1 Introduction

Mangoes ( *Mangifera indica* L.) are perishable tropical fruits due to fast ripening, disease vulnerability, and cold sensitivity below 10°C–13°C (Mogollón et al. 2020). Shelf life is reduced by water loss, texture and color changes, damage, and attacks by microorganisms, notably *Colletotrichum gloeosporioides* (Sharma et al. 2021). Strategies to extend shelf life include refrigeration, atmospheric modification, heat treatment, coatings, and fungicides (Xing et al. 2019). However, the use of synthetic fungicides carries risks for human health and the environment while also contributing to pathogen resistance (Nesa Chowdhury et al. 2022). Edible coatings offer a sustainable alternative, delaying ripening, improving appearance, and extending shelf life without toxicity or pollution at low costs (Asghari et al. 2022; Panahirad, Naghshiband‐Hassani, and Mahna 2020). Pectin, a cost‐effective, biodegradable polysaccharide, is ideal for coatings due to its gel‐forming capability and gas barrier properties (Pérez Espitia et al. 2014). Pectin coatings can also encapsulate antioxidants, flavorings, and antimicrobial agents, enhancing food protection against microbes (Sucheta et al. 2019). Incorporating natural antimicrobial agents such as essential oils can improve the functionality of coatings and protect food from microbiological damage (Perumal et al. 2022; Prasad et al. 2024).

Essential oils offer a natural alternative to synthetic preservatives, meeting consumer demands for products with minimal pesticide residues (Nesa Chowdhury et al. 2022). Incorporating essential oils into edible coatings mitigates their direct application drawbacks like intense aromas or phytotoxicity, ensuring controlled release and enhanced barrier functions (Palou et al. 2016). Oregano essential oil (OEO), noted for its potent antimicrobial properties, owes its effectiveness to carvacrol and thymol. These compounds disrupt fungal cell membranes by hindering ergosterol synthesis and increasing membrane porosity (Chailot et al. 2015).

The coating composition crucially impacts its effect on fruits. Selecting appropriate coating materials and optimal concentration depends on the physiology of the studied fruit (Elawady, Sallam, and Abd El‐Salam 2022).

The study aims to develop a pectin–glycerol–OEO coating to control the postharvest deterioration of mangoes through a three‐step process: (1) optimization using a mixture design to examine the influence of coating components on respiration rate, quality parameters, and anthracnose incidence in stored Keitt mangoes, (2) fitting cubic models representing the behavior of quality parameters in coated mangoes, and (3) validating the optimized coating solution and assessing its effectiveness in controlling postharvest mango deterioration compared to untreated mangoes and those treated with a commercial fungicide.

## 2 Materials and Methods

### 2.1 Materials

Keitt mangoes at ripeness stage 2 (10%–11% soluble solids), selected for size, shape, color, and absence of damage, were used. *Colletotrichum gloeosporioides* strains isolated from anthracnose‐affected mangoes were employed. Coating solution materials included high‐methoxy citric pectin (Tecnas, Medellín, Colombia), glycerol (JT Baker, Mexico), and oregano ( *Origanum vulgare* L.) essential oil (NOW foods, United States). Prochloraz (0.12% w/v; TCI America, Portland, OR, USA), a commercial fungicide, served as a comparison treatment for validation experiments.

### 2.2 Coating Solutions

#### 2.2.1 Coating Solutions Preparation

Coating solutions were made by dissolving high‐methoxy citric pectin in distilled water at 70°C for 2 h with a mechanical stirrer (Heidolph, Germany) until fully dissolved. The pectin solution was cooled to room temperature (25°C). Subsequently, the corresponding amounts of glycerol and OEO were added with constant stirring. For the dispersion of the oil droplets in the solution, a homogenizer (Ultra Turrax IKA T‐25, Germany) was used at a speed of 12,000 rpm for 3 min (Sánchez‐Tamayo, Ochoa‐Martínez, and Critzer 2021).

#### 2.2.2 Viscosity of Coating Solutions

The apparent viscosity of the forming solutions was determined in a viscometer (Brookfield, DVIII Ultra, United States) equipped with a No. 21 needle and temperature control (PolyScience, United States). A forming solution of 15 mL was used at 25°C. Measurements were performed at 100 rpm for 2 min according to the procedure described by Saberi et al. (2016).

#### 2.2.3 In Vitro Antifungal Activity

The in vitro antifungal activity (IAA) of the coating‐forming solutions was determined using a disk diffusion test (Balouiri, Sadiki, and Ibnsouda 2016). Conidial suspensions were obtained from *C. gloeosporioides* strains at 10 days' incubation in potato–dextrose–agar (PDA) medium and adjusted to 10^5^ CFU/mL. PDA medium in Petri dishes was inoculated with 0.1 mL spore suspension. Each coating solution (10 mL) was added to 6 mm filter paper disks (OXOID, United States) placed centrally on the inoculated medium surface. After sealing, dishes were incubated for 7 days at 25°C. Inhibition zones around the disks were measured (cm^2^) using ImageJ software to determine the antifungal activity.

### 2.3 Fruit Treatment and Evaluation

The mangoes were immersed in 2 L of forming solution for 1 min, dried for 3 h at 25°C, and stored in an environmental chamber (Dártico, Cali, Colombia) for up to 12 days at 14°C ± 2°C and 85% ± 2% of relative humidity (RH).

#### 2.3.1 Respiration Rate

The respiration rate (O_2_ consumption and CO_2_ production) was determined using a respirometer equipment (Ortega Villalba et al. 2014) adjusted with an infrared CO_2_ sensor (COZIR W20, United States) and an O_2_ fluorescence sensor (LuminOx, United Kingdom). Four mangoes were used per treatment. The test was performed for 50 min, with measurements every 10 s. The respiration rate was determined using Equations (1) and (2).

(1) $${\mathrm{RR}}_{\left(O_{2}\right)}=\frac{R_{o_{2}}\;V_{f}}{w∆t}\times 1.31$$

(2) $${\mathrm{RR}}_{\left(O_{2}\right)}=\frac{R_{co_{2}}\;V_{f}}{w∆t}\times 1.8$$

where RR is the respiration rate (mg/kg h) measured as O_2_ consumption or CO_2_ production, $R_{o_{2}}$ is the change in O_2_ concentration (ppm), $R_{co_{2}}$ is the change in CO_2_ concentration (ppm), $V_{f}$ is the free volume of the container (m^3^), *w* is the weight of the fruit (kg), Δ*t* is the reading interval (h), 1.31 is the conversion factor from ppm of O_2_ to mg O_2_/m^3^, and 1.8 is the conversion factor from ppm of CO_2_ to mg CO_2_/m^3^.

#### 2.3.2 Quality Parameters

Firmness was determined by puncture in the equatorial zone of the skinless mango via a uniaxial compression texturometer (EZ‐Test, Shimadzu, United States) according to Khaliq et al. (2016). A 2 mm diameter flat‐tipped cylindrical probe, 60 mm/min speed, and 10 mm penetration were used. Soluble solids (SS, %) were measured using a digital refractometer (Atago RX‐700α, Japan) on 10 g filtered mango pulp. Weight loss was determined relative to the initial weight. Pulp color in the equatorial fruit zone was measured using a Colorflex Hunter Lab colorimeter (Reston, USA) with CIE Lab* coordinates.

#### 2.3.3 Natural Incidence of Anthracnose

The natural incidence of anthracnose (NIA) was determined by counting the diseased fruits at storage time according to Equation (3) (Jongsri et al. 2016). The disease was identified by the presence of irregular black or brown spots characteristic of anthracnose in mangoes.

(3) $$\mathrm{NIA}\;\left(\%\right)=\frac{\text{Number of disease fruits}}{\text{Number of total fruits}}\times 100$$

#### 2.3.4 Microstructure of the Coated Mango

The surface microstructures of the coated mangoes were analyzed under an environmental scanning electron microscope (ESEM) (FEI QUANTA 250, United States). The samples were observed at 14 kV and a working distance of 9–12 mm.

### 2.4 Experimental Design

The study employed an extreme vertices mixture design within response surface methodology (RSM), setting constraints on proportions of pectin, glycerol, and OEO, as shown in Equation (4).

(4) $$0\leq a_{i}\leq x_{i}\leq b_{i}\leq 1$$

where $a_{i}$ is the lower constraint and $b_{i}$ is the upper constraint for the component $x_{i}$ of the mixture (Gutierrez and De la Vara 2008).

Component restrictions were set at 3.0%–4.5% w/v for pectin, 0.25%–0.75% w/v for glycerol, and 0%–1.75% w/v for OEO, with a total sum of 5% to ensure coating homogeneity, based on preliminary OEO sensory acceptance tests. The design included axial points for curvature estimation and five central point replicates for error and repeatability analyses, which are critical for assessing the statistical significance of coefficients resulting in 15 experimental runs conducted randomly to eliminate systematic errors. Fifteen experimental runs with five replicates (central points) and three mangoes per replicate were used. Mixture compositions are detailed in Table S1.

### 2.5 Mathematical Model and Statistical Analysis

The experimental data were fitted to a special cubic model according to Equation (5).

(5) $$Y_{i}=\beta_{1}X_{1}+\beta_{2}X_{2}+\beta_{3}X_{3}+\beta_{12}X_{1}X_{2}+\beta_{13}X_{1}X_{3}+\beta_{23}X_{2}X_{3}+\beta_{123}X_{1}X_{2}X_{3}$$

where $Y_{i}$ is the response variable, *β*_*i*_ is an estimation coefficient, and *X*_1_ (pectin), *X*_2_ (glycerol), and *X*_3_ (OEO) are the independent variables.

Analysis of variance (ANOVA) was utilized to assess the fit of the model and the importance of various factors on the dependent variable, considering the total squares' sum and the residual squares' sum. These statistical evaluations were conducted using MINITAB software version 19.

The correlation between the theoretical model and experimental data was evaluated through regression coefficients, *R* ^2^, and adjusted *R* ^2^, where values close to 1 indicate a strong correlation between model predictions and experimental observations. The root‐mean‐square error (RMSE) evaluated the predictive accuracy of the model, with lower RMSE values denoting greater precision. These parameters, R^2^, adjusted R^2^, and RMSE, are shown in Equations (6), (7), and (8), respectively.

(6) $$R^{2}=1-\frac{\sum_{i=1}^{n}{\left(y-\hat{y}\right)}^{2}}{\sum_{i=1}^{n}{\left(y-\bar{y}\right)}^{2}}$$

(7) $$\text{Adjusted}\;R^{2}=1-\frac{\left(1-R^{2}\right)\left(n-1\right)}{n-P-1}$$

(8) $$\text{RMSE}=\sqrt{\frac{\sum_{i=1}^{n}{\left(y-\hat{y}\right)}^{2}}{n}}$$

where $\hat{y}$ is the observed value, $\hat{y}$ is the predicted value, *n* is the number of observations, and *p* is the number of predictors.

### 2.6 Optimization

A response optimizer was used to find the optimal mixture through composite desirability, aiming to minimize O_2_ consumption, CO_2_ production, weight loss, and anthracnose incidence while maximizing pulp brightness and firmness, assessed on day 12. All variables were equally weighted. The selection was based on two criteria: (1) relevance of quality parameters for mangoes during storage and (2) response variable models explaining over 70% of behavior (*R* ^2^ adj. > 0.7), according to Gutierrez and De la Vara (2008).

### 2.7 Experimental Validation

Validation experiments were carried out by applying the optimal coating to Keitt mangoes, followed by storage for 12 days at 14°C and 85% RH. The observed responses were compared with the optimal predictions from Section 2.6, using relative error (RE), as shown in Equation (9).

(9) $$\mathrm{RE}=\frac{y-\hat{y}}{y}\;100$$

The effectiveness of the optimal coating was evaluated against a commercial fungicide (Prochloraz) and untreated mangoes (immersed in distilled water), stored for 18 days at 14°C and 85% RH. Parameters including respiration rate, firmness, soluble solids, pulp brightness, and natural anthracnose incidence were assessed according to Section 2.3 methodologies. Three treatments with two replicates and three samples per replicate were used. All results were reported as means ± standard errors. Statistical analysis used one‐way ANOVA at *p* < 0.05 significance, with the Tukey test for treatment differences, using Minitab v.19.

## 3 Results and Discussion

### 3.1 Viscosity and In Vitro Antifungal Activity of Coating Solutions

Table S2 presents the ANOVA and coefficients for the cubic models of viscosity (μ) and IAA in coating solutions, with detailed equations in Table S3. Table S2 lists the coefficients for linear terms X_1_, X_2_, and X_3_, using an asterisk (*) to denote that *p*‐values for these terms are not calculated in mixture designs due to multicollinearity from the requirement that components sum to 100%. This limitation prevents the independent evaluation of the linear effect of each component. The analysis is on the significance of higher‐order terms, considering linear effects are already incorporated, without subjecting them to hypothesis testing like interactions or quadratic effects.

For the quadratic terms representing two‐component interactions, only X_1_ X_3_ is statistically significant for IAA (*p*‐value: 0.032), and X_2_ X_3_ shows a strong effect on IAA (*p*‐value: 0.000), indicating significant antifungal activity without necessarily affecting viscosity. The three‐component interaction (X_1_ X_2_ X_3_) is not significant for viscosity (*p*‐value: 0.491) but impacts IAA. Table S4 details the special cubic model's observed and predicted values for viscosity and IAA, including *R* ^2^, adjusted *R* ^2^, and RMSE. The analysis reveals that pectin X_1_, X_2_, and X_3_ interactions are well‐explained by special cubic models, with adjusted *R* ^2^ between 97.53% and 99.22%.

Figure S1 presents contour plots for (a) viscosity and (b) IAA. Figure S1a shows that the higher the OEO concentration, the lower the solution viscosities, attributable to less pectin in higher OEO concentration solutions at the same total solids, causing slightly less thickening and less viscous coatings (Tonon, Grosso, and Hubinger 2011). Viscosity significantly affects coating adherence to fruit surfaces (Wigati et al. 2023). High viscosity indicates dipping method suitability; low viscosity suggests spraying suitability for edible coatings.

Figure S1b shows that the effectiveness of treatments in preventing fungal growth, measured by inhibition zone size, was linked to OEO concentration in the coating solution. Zones larger than 20 cm^2^ were seen for OEO concentrations over 1%. OEO contained 75.9% carvacrol and 4.9% thymol (Sánchez‐Tamayo, Ochoa‐Martínez, and Critzer 2021), monoterpenes known to hinder ergosterol biosynthesis, create pores in microorganism membranes, and induce hyphal morphological changes via penetration into membranes (Chailot et al. 2015).

### 3.2 Respiration Rate and Quality Parameters of Coated Mangoes

Table S5 details ANOVA and coefficients for cubic models on the quality parameters of coated mangoes, with equations in Table S6. Coefficients for linear terms X_1_, X_2_, and X_3_ lack *p*‐values, as explained in Section 3.1. Quadratic terms like X_1_X_2_ and X_1_X_3_ influence RRO_2_ positively, indicating enhancement, while X_1_X_2_ decreases firmness, suggesting softening. The cubic term X_1_X_2_X_3_ is significant for RRO_2_ and NIA (*p*‐values: 0.00), showing a strong effect of combining X_1_, X_2_, and X_3_, notably decreasing RRO_2_ and increasing the L* value (*p*‐value: 0.01).

Table 1 presents the observed and predicted values for RRO_2_, RRCO_2_, firmness, SS, WL, L*, and NIA, alongside *R* ^2^, adjusted *R* ^2^, and RMSE, indicating good model fits. *R* ^2^ values span from 82.42% (RRCO_2_) to 97.87% (NIA), demonstrating the explanatory power of the model. Adjusted *R* ^2^ values show strong fits as well, with the lowest at 69% (SS) and the highest at 96.85% (NIA). RMSE values, ranging from 0.12% (SS) to 2.09% (RRO_2_), confirm the model's accuracy in predicting response variables.

**TABLE 1:** Observed and predicted values for respiration rate (RRO_2_, RRCO_2_), SS, WL, L*, and NIA of coated mangoes.

Exp number (*n*)	Coating solutions			RRO_2_ (mg/kg h)		RRCO_2_ (mg/kg h)		Firmness (N)		SS (%)		WL (%)		L*		NIA (%)	
	Pectin (%) (w/v)	Glycerol (%) (w/v)	OEO (%) (w/v)	Obs.	Fit Cubic model	Obs.	Fit Cubic model	Obs.	Fit Cubic model	Obs.	Fit Cubic model	Obs.	Fit Cubic model	Obs.	Fit Cubic model	Obs.	Fit Cubic model
1	3.00	0.25	1.75	39.35	40.18	35.74	35.41	25.15	25.59	13.12	13.02	3.78	3.79	79.51	79.58	23.50	22.98
2	3.00	0.85	1.15	22.64	24.01	33.98	33.51	24.50	24.89	12.15	11.91	3.61	3.62	78.04	78.23	22.10	21.11
3	4.50	0.25	0.25	11.44	12.59	28.84	28.34	35.20	38.50	11.95	12.53	3.15	3.21	83.24	82.56	18.70	18.94
4	4.50	0.50	0.00	22.01	21.85	24.74	24.14	35.31	34.78	10.38	10.44	2.89	2.91	82.84	82.92	28.20	26.75
5	4.15	0.85	0.00	32.14	33.20	26.46	28.17	34.12	34.93	11.63	11.57	3.16	3.21	80.50	80.02	29.30	29.28
6	3.00	0.55	1.45	34.99	33.35	29.85	30.74	22.00	21.71	11.02	11.35	3.66	3.65	80.16	79.81	21.00	22.40
7	3.75	0.25	1.00	32.25	30.77	31.68	31.47	34.18	32.22	14.35	14.23	4.04	3.99	79.90	80.28	15.40	15.52
8	4.32	0.68	0.00	30.10	29.40	27.04	24.88	33.21	33.66	10.43	10.66	3.11	3.06	81.37	81.74	27.80	28.51
9	4.50	0.38	0.13	16.92	15.57	23.22	25.02	39.45	35.70	12.02	11.27	3.07	2.99	82.54	83.15	21.20	22.07
10	3.58	0.85	0.58	17.76	15.42	26.20	25.77	29.01	27.23	12.23	12.38	3.20	3.14	80.55	80.81	14.10	14.88
11	3.83	0.54	0.63	16.88	20.12	25.78	22.90	27.10	26.92	11.30	12.02	3.32	3.28	82.83	82.36	15.00	14.67
12	3.83	0.54	0.63	16.27	20.12	18.87	22.90	23.90	26.92	12.53	12.02	3.32	3.28	81.97	82.36	15.20	14.67
13	3.83	0.54	0.63	23.81	20.12	24.84	22.90	26.50	26.92	12.68	12.02	3.04	3.28	82.69	82.36	14.00	14.67
14	3.83	0.54	0.63	18.08	20.12	20.71	22.90	29.20	26.92	12.02	12.02	3.07	3.28	82.76	82.36	14.70	14.67
15	3.83	0.54	0.63	22.31	20.12	24.03	22.90	25.00	26.92	11.63	12.02	3.56	3.28	82.00	82.36	15.60	14.67
*R* ^2^ (%)				92.94		84.22		87.4		82		85.97		92.93		97.87	
*R* ^2^‐adj (%)				87.65		72.39		78		69		75.44		87.63		96.28	
RSME (%)				2.09		1.77		1.86		0.41		0.12		0.40		0.77	

#### 3.2.1 Respiration Rate

Mangoes coated with solutions 1, 2, and 6, which had high OEO levels (> 1%), showed the highest respiration rates, as indicated in Table 1. This study found that OEO concentrations above 1% damaged mango skin (see Figure S2), increasing respiration. Carvacrol in OEO may damage skin cells, accelerating metabolism and causing premature ripening (Guerreiro et al. 2015).

Figure S3a,b shows that coating solutions without OEO or with high OEO (> 1%) had RRO_2_ and RRCO_2_ values > 20 mg/kg h, while high pectin and glycerol decreased these. According to Tavassoli‐Kafrani et al. (2022), lowering O_2_ and increasing CO_2_ benefit mango quality preservation. An ideal coating should allow respiratory gas exchange while slowing respiration without fermentation. The findings of this study showed that the mean concentrations of pectin, glycerol, and OEO caused medium/low O_2_ consumption and CO_2_ production, delaying ripening and increasing coated mango shelf life.

#### 3.2.2 Firmness

Table 1 shows firmness values between 22 and 39.45 N on storage day 12. Figure S4a reveals that high concentrations of pectin and glycerol maintained the firmness above 30 N. Polysaccharide coatings help maintain firmness in avocados, mangoes, bananas, and minimally processed fruits (Bello‐Lara et al. 2016; Hoa and Ducamp 2008). Conversely, increasing OEO concentration decreased pulp firmness due to the increased respiration rate.

#### 3.2.3 Soluble Solids

The SS of the coated mangoes presented values between 10.38% and 14.35% on day 12 of storage (Table 1). According to the contour plot (Figure S4b), lower SS (11%–12%) values were obtained with the mean concentrations of the components in the coating solutions. These SS values are an adequate indicator of maturity for transporting and commercializing fruits. However, the minimum acceptable level of SS may differ for mangoes destined for export depending on the transport distance (Liu, Wang, and Young 2014).

#### 3.2.4 Weight Loss

Table 1 shows 2.89%–4.04% WL values on storage day 12. Figure S4c revealed greater WL for high OEO coating concentrations, related to the undesired effects of > 1% OEO on respiration rate and firmness. Coatings with mean component concentrations reduced WL to < 3%, showing their interaction slowed fruit transpiration effectively. Consistent with Moalemiyan, Ramaswamy, and Maftoonazad (2012), polysaccharide coatings alone minimally influence WL, while adding a lipid improves effectiveness by regulating the hydrophilic–hydrophobic balance, restricting water loss.

#### 3.2.5 Pulp Brightness

The lightness (L*) values for mango pulp ranged from 78.04 to 83.24 after 12 days of storage. Figure S4d shows that compared to coatings with lower pectin concentrations, those with higher pectin concentrations reduce changes in luminosity, resulting in brighter colored pulp.

#### 3.2.6 Natural Incidence of Anthracnose

Table 1 shows coatings with OEO addition reduced anthracnose incidence, confirming its antifungal effect. Figure S4e shows the lowest incidence (NIA 15%–20%) at mean component concentrations, indicating component interactions besides the antifungal effect of OEO contributed to decreasing this variable. According to Jongsri et al. (2016), surface film formation can maintain OEO‐active compounds over time and create a low O_2_/high CO_2_‐modified atmosphere unfavorable for microbial growth.

In contrast to *in vitro* antifungal tests (Table S4), the highest OEO concentrations (> 1%) did not decrease anthracnose incidence. This behavior could be due to the local phytotoxic effect of high concentrations of essential oils, where the main components affect fruit tissue, softening it for easier germinated spore penetration (Figure S2). Similar results were reported for papaya coatings with thyme/Mexican lime oils and tomato coatings with OEO/thyme/lemongrass/coriander oils by Bosquez‐Molina et al. (2010) and Plotto, Roberts, and Roberts (2003).

### 3.3 Optimization

**FIGURE 1:**
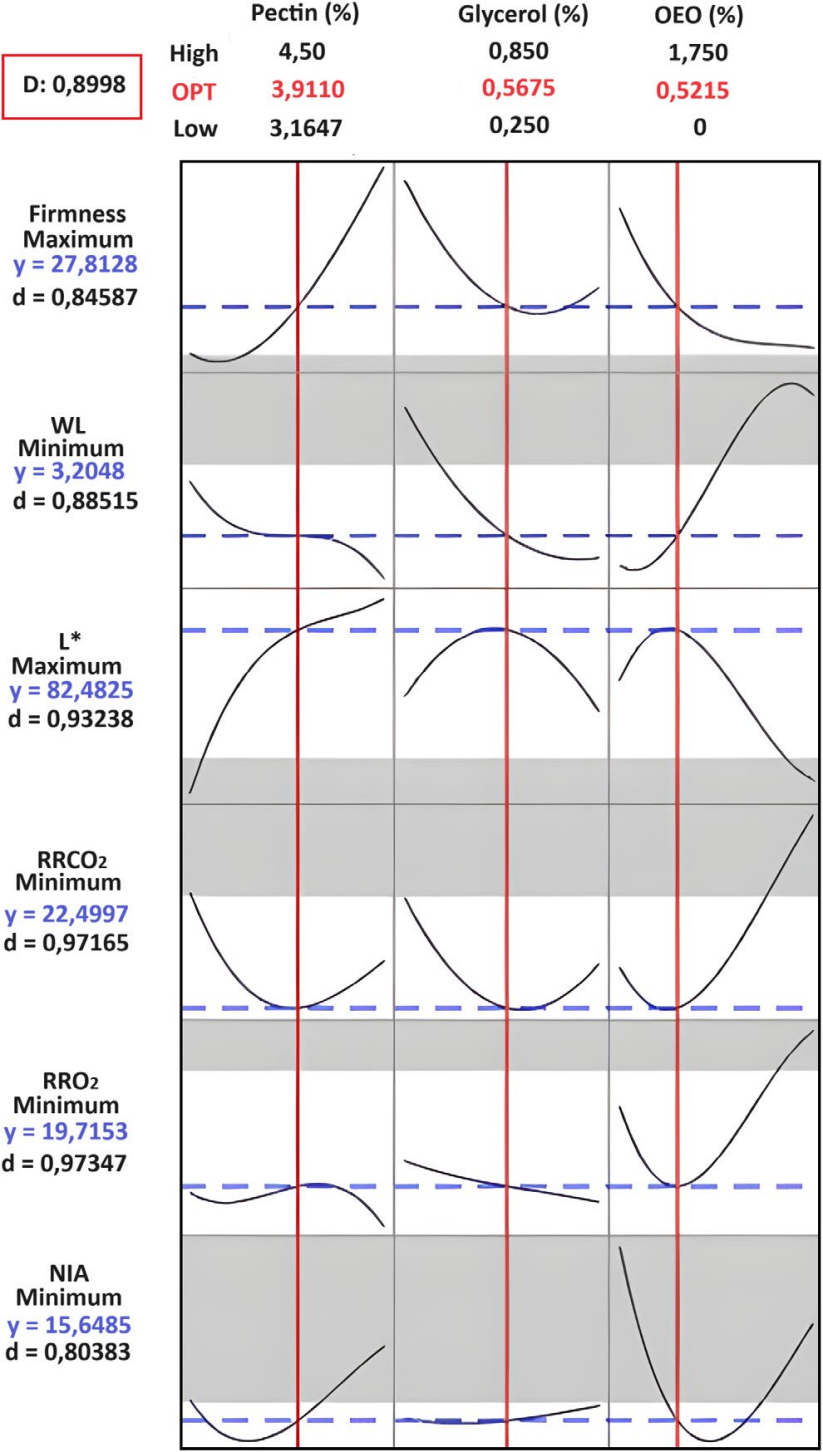
Response optimization plot showing the optimized coating.

Figure 1 presents the optimization results, showing a coating solution of 3.91% pectin, 0.57% glycerol, and 0.52% OEO with a composite desirability of 0.8998 which are highly satisfactory (Dave, Rao, and Nandane 2016). The optimal coating yielded a firmness of 27.82 N, a weight loss of 3.2%, a pulp brightness of 82.48, a respiration rate of 22.50 mg CO_2_/kg h, a respiration rate of 19.72 mg O_2_/kg h, and an anthracnose incidence of 15.65%.

### 3.4 Model Validation

Table 2 presents the experimental data and those predicted by the models after 12 days of storage at 14°C and 85% RH. The recorded values for all parameters fall within the 95% prediction interval and closely align with the predicted values, suggesting the reliability of the models. The relative error varied between 0.88% and 12.28%, indicating an adequate precision of the methodology developed for the optimization process and validity of the responses obtained with the experimental design.

**TABLE 2:** Model validation for RRCO_2_, RRO_2_, WL, L*, firmness, and NIA of mango treated with the optimized coating solution.

Parameter	Experimental value	Predicted value	Relative error (%)
Respiration rate O_2_ (mg O_2_/m^3^ kg h)	18.19 ± 0.91	19.72	8.41
Respiration rate CO_2_ (mg CO_2_/m^3^ kg h)	23.31 ± 0.51	22.49	3.51
Weight loss (%)	2.85 ± 0.33	3.20	12.28
Lightness (L*)	83.21 ± 0.47	82.48	0.88
Firmness (N)	28.85 ± 1.07	27.82	3.63
Anthracnose incidence (%)	14.15 ± 0.24	15.64	10.53

### 3.5 Efficacy of the Optimal Coating

#### 3.5.1 Microstructure of Coated Mango

Figure S5a shows the micrographs of mango skin coated with the optimized solutions. The coated skin has a smooth, continuous appearance, whereas the uncoated skin is rough and corrugated, indicating an effective application (Moore‐Neibel et al. 2013). Figure S5b shows the coating uniformly covers the fruit skin stomata, generating a gas exchange barrier between the fruit and environment, decreasing the respiration rate, and changing the mango quality characteristics during storage.

#### 3.5.2 Respiration Rate and Quality Parameters

Figure S6 shows the respiration rate, firmness, SS, lightness, and NIA results for mangoes with the optimized coating compared to Prochloraz treatment and untreated mangoes. Figure S6a demonstrates that coated mangoes exhibited reduced CO_2_ production after 6 storage days compared to Prochloraz‐treated and untreated mangoes (*p*‐value: 0.013), suggesting effective respiration inhibition, 20% less than control. Figure S6b shows a gradual pulp firmness decrease during storage without treatment differences until day 9. However, coated mangoes had significantly higher firmness (*p*‐value: 0.028) on day 12, with 25–30 N values until day 18. Firmness > 26 N indicates the best maturity for shipping mangoes (Brecht 2015).

Figure S6c shows SS values for all treatments started between 10% and 12% (day 0: mature green) and gradually increased until day 18. Mangoes with optimal coating maintained 13%–14% SS at the storage end, whereas Prochloraz‐treated and untreated mangoes had 15%–17% SS. SS > 15% indicates consumption maturity, so the optimal coating contributed to delaying ripening (Brecht 2015).

A pulp brightness (L*) decrease was observed over time for all samples (Figure S6d). Coated mangoes maintained brightness throughout the 18‐day storage, with significantly higher values (*p*‐value: 0.018) than Prochloraz‐treated and untreated mangoes. Coatings alter the internal atmosphere of the fruit, preserving the green color and delaying mesocarp discoloration (Maftoonazad et al. 2007). Prochloraz‐treated and untreated mangoes exhibited increased color variations and pulp darkening from light yellow to orange by storage end. Prochloraz‐treated and untreated mangoes exhibited increased color variations and pulp darkening from light yellow to orange by storage end.

Figure S6e shows that the optimal coating reduced anthracnose incidence by up to 30% compared to untreated mangoes. Equal anthracnose control effectiveness was observed between optimal coating and Prochloraz after 18 days of storage. Sensory evaluation conducted in a subsequent study showed that the optimal coating had no impact on the sensory characteristics of mango (aroma, flavor, appearance, and acceptance) (Sánchez‐Tamayo, Ochoa‐Martínez, and Critzer 2021).

## 4 Conclusions

The mixture design approach enables to develop cubic models that accurately forecast the respiration rate and quality attributes of coated mangoes. The optimal coating acts as a barrier for the exchange of gases between the fruit and the environment. The barrier formed by the coating, as well as the interactions between the three components of the coating, reduced the respiration and ripening process of coated mangoes and controlled the natural incidence of anthracnose with the same effectiveness as Prochloraz, thus providing an alternative to synthetic fungicides. These findings underscore the potential of the proposed coating formulation for enhancing postharvest preservation and quality of Keitt mangoes, presenting a sustainable and potential viable alternative for fruit preservation practices.

## Author Contributions

**Martha Sanchez‐Tamayo:** conceptualization, methodology, investigation, writing – reviewing and editing. **José Luis Plaza‐Dorado:** writing, statistical analysis, review, and editing. **Claudia Ochoa‐Martínez:** review and editing, validation, and supervision. All authors have read and agreed to the published version of the manuscript.

## Ethics Statement

This study does not involve any human or animal testing.

## Conflicts of Interest

The authors declare no conflicts of interest.

## Supporting information


Data S1.

## Data Availability

The authors have nothing to report.
