# Supplementary material for: Influence of Composite Edible Coating of Pectin, Glycerol, and Oregano Essential Oil on Postharvest Deterioration of Mango Fruit
Source: Food Sci Nutr. 2024 Nov 21;12(12):10646–54. doi: 10.1002/fsn3.4545 (PMC11666998; doi:10.1002/fsn3.4545)
Supplement: Supplementary file 1 — Data S1. [file FSN3-12-10646-s001.docx]

**Development of an edible coating based on pectin, glycerol, and oregano essential oil for controlling mango postharvest deterioration**

Supplementary material

Martha Sanchez-Tamayo^1*^José Luis Plaza-Dorado^2^, Claudia Ochoa-Martínez^2^

^1^ Faculty of Agronomic Engineering, University of Tolima, Ibagué, Colombia

^2^ School of Food Engineering, University of Valle, Cali, Colombia

* Corresponding author: misanchezt@ut.edu.co

Table S1. Extreme vertices design for three component mixture of pectin, glycerol and OEO

| **Exp. Number** | **Pectin (%) (w/v)** | **Glycerol (%) (w/v)** | **OEO**  **(%) (w/v)** |
| --- | --- | --- | --- |
| 1 | 3.00 | 0.25 | 1.75 |
| 2 | 3.00 | 0.85 | 1.15 |
| 3 | 4.50 | 0.25 | 0.25 |
| 4 | 4.50 | 0.50 | 0.00 |
| 5 | 4.15 | 0.85 | 0.00 |
| 6 | 3.00 | 0.55 | 1.45 |
| 7 | 3.75 | 0.25 | 1.00 |
| 8 | 4.32 | 0.68 | 0.00 |
| 9 | 4.50 | 0.38 | 0.13 |
| 10 | 3.58 | 0.85 | 0.58 |
| 11 | 3.83 | 0.54 | 0.63 |
| 12 | 3.83 | 0.54 | 0.63 |
| 13 | 3.83 | 0.54 | 0.63 |
| 14 | 3.83 | 0.54 | 0.63 |
| 15 | 3.83 | 0.54 | 0.63 |

**Table S2**. ANOVA and coefficients terms of the special cubic models for viscosity (µ) and IAA of the coating solutions.

| **Source** | **Term** | **µ (Pa s)** | | **IAA (cm^2^)** | |
| --- | --- | --- | --- | --- | --- |
|  |  | ***Coefficient value*** | ***p-Value*** | ***Coefficient value*** | ***p-Value*** |
| Linear | *X_1_* | 141.715 | * | -1.203 | * |
|  | *X_2_* | 220.926 | * | -28.729 | * |
|  | *X_3_* | 9.726 | * | -8.84 | * |
| Quadratic | *X_1_ X_2_* | 120.612 | 0.418 | 8.492 | 0.437 |
|  | *X_1_ X_3_* | -56.215 | 0.378 | 11.453 | 0.032 |
|  | *X_2_ X_3_* | 293.684 | 0.518 | 203.166 | 0.00 |
| Special Cubic | *X_1_ X_2_ X_3_* | 85.463 | 0.491 | -63.79 | 0.00 |

**Table S3**. Special cubic regression models for parameters of coatings and film-forming solutions

| **Response** | **Cubic regression models** |
| --- | --- |
| **Viscosity** | μ = 141.715X_1_ + 220.926X_2_+ 9.726X_3_ -120.612X_1_X_2_ - 56.215X_1_X_3_ + 293.684X_2_X_3_ -85.463X_1_X_2_X_3_ |
| ***In vitro* inhibition area** | IA = -1.203X_1_ - 28.729X_2_ - 8.846X_3_+ 8.492 X_1_X_2_+ 11.453X_1_X_3_+ 203.166X_2_X_3_ - 63.79 X_1_X_2_X_3_ |

**Table S4**. Observed and predicted values for viscosity (µ) and IAA of the coating solutions

| **Exp. number (n)** | **Coating solutions** | | | **µ (Pa s)** | | **IAA (cm^2^)** | |
| --- | --- | --- | --- | --- | --- | --- | --- |
|  | **Pectin (%) (w/v)** | **Glycerol (%) (w/v)** | **OEO (%) (w/v)** | **Obs.** | **Fit Cubic model** | **Obs.** | **Fit Cubic model** |
| 1 | 3 | 0.25 | 1.75 | 127.17 | 128.13 | 45.13 | 45.39 |
| 2 | 3 | 0.85 | 1.15 | 157.67 | 159.07 | 34.22 | 34.49 |
| 3 | 4.5 | 0.25 | 0.25 | 498.4 | 490.77 | 0.91 | 2.39 |
| 4 | 4.5 | 0.5 | 0 | 496 | 476.80 | 0 | -0.67 |
| 5 | 4.15 | 0.85 | 0 | 365 | 350.45 | 0 | 0.54 |
| 6 | 3 | 0.55 | 1.45 | 150.17 | 146.96 | 41.22 | 41.00 |
| 7 | 3.75 | 0.25 | 1 | 261.5 | 265.81 | 22.41 | 21.36 |
| 8 | 4.32 | 0.68 | 0 | 384 | 409.93 | 0 | 0.20 |
| 9 | 4.5 | 0.38 | 0.13 | 466 | 482.37 | 0.9 | -0.45 |
| 10 | 3.58 | 0.85 | 0.58 | 206.17 | 212.16 | 4.44 | 3.38 |
| 11 | 3.83 | 0.54 | 0.63 | 258.5 | 271.66 | 3.64 | 5.51 |
| 12 | 3.83 | 0.54 | 0.63 | 299.83 | 271.66 | 5.77 | 5.51 |
| 13 | 3.83 | 0.54 | 0.63 | 272.83 | 271.66 | 7.31 | 5.51 |
| 14 | 3.83 | 0.54 | 0.63 | 280 | 271.66 | 4.5 | 5.51 |
| 15 | 3.83 | 0.54 | 0.63 | 257.5 | 271.66 | 4.71 | 5.51 |
| ***R^2^*** | | | | **98.59** | | **99.56** | |
| ***R^2^-adj*** | | | | **97.53** | | **99.22** | |
| ***RSME*** | | | | **13.86** | | **1.02** | |

**Table S5**. ANOVA and coefficients terms of the special cubic models for Firmness, soluble solids (SS), Water loss (WL), Brightness (L*) and Natural Incidence of Anthracnose (NIA)

| ***Source*** | ***Term*** | **RRO_2_ (mg/kgh)** | | **RRCO_2_ (mg/kgh)** | | **Firmness (N)** | | **SS (%)** | | **WL (%)** | | **L*** | | **NIA (%)** | |
| --- | --- | --- | --- | --- | --- | --- | --- | --- | --- | --- | --- | --- | --- | --- | --- |
|  |  | ***Coef.***  ***value*** | ***p-value*** | ***Coef.***  ***value*** | ***p-value*** | ***Coef.***  ***value*** | ***p-value*** | ***Coef.***  ***value*** | ***p-value*** | ***Coef.***  ***value*** | ***p-value*** | ***Coef.***  ***value*** | ***p-value*** | ***Coef.***  ***value*** | ***p-value*** |
| Linear | *X_1_* | -4.07 | * | 7.20 | * | 10.22 | * | 2.72 | * | 0.52 | * | 16.66 | * | 3.24 | * |
|  | *X_2_* | -194.78 | * | 170.15 | * | 152.72 | * | 47.19 | * | 2.16 | * | -23.64 | * | -48.87 | * |
|  | *X_3_* | -54.86 | * | -4.44 | * | -9.68 | * | -4.61 | * | -3.53 | * | 26.35 | * | 8.69 | * |
| Quadratic | *X_1_ X_2_* | 61.13 | 0.02 | -41.48 | 0.05 | -38.93 | 0.07 | -11.29 | 0.03 | -0.22 | 0.86 | 8.77 | 0.06 | 16.26 | 0.07 |
|  | *X_1_ X_3_* | 27.65 | 0.02 | 5.37 | 0.51 | 3.82 | 0.65 | 2.86 | 0.15 | 1.58 | 0.01 | -4.12 | 0.04 | -0.70 | 0.84 |
|  | *X_2_ X_3_* | 252.21 | 0.01 | 31.82 | 0.58 | 2.79 | 0.96 | -9.12 | 0.50 | 7.87 | 0.06 | -22.40 | 0.11 | 111.61 | 0.00 |
| Special  Cubic | *X_1_ X_2_ X_3_* | -79.43 | 0.00 | -24.37 | 0.15 | -14.02 | 0.40 | -1.10 | 0.76 | -2.83 | 0.02 | 10.83 | 0.01 | -35.88 | 0.00 |

**Table S6**. Cubic regression models for quality parameters of coated mango

| **Response** | **Cubic regression models** |
| --- | --- |
| **RR O_2_** | RRO_2_ = 4.067X_1_ - 194.84X_2_ -54.835X_3_ + 61.145X_1_X_2_ + 27.639X_1_X_3_ + 252.203X_2_X_3_ -79.425X_1_X_2_X_3_ |
| **RR CO_2_** | RRCO_2_ = 7.202 X_1_ + 170.116 X_2_ - 4.433 X_3_ -41.479 X_1_X_2_ + 5.372 X_1_X_3_ + 31.863 X_2_X_3_ -24.382 X_1_X_2_X_3_ |
| **Firmness** | F = 4.099 X_1_ - 343.631 X_2_ - 34.278 X_3_+ 84.862 X_1_X_2_ + 7.56 X_1_X_3_ + 306.311 X_2_X_3_ -67.781 X_1_X_2_X_3_ |
| **Soluble solids** | SS = 2.723 X_1_ + 47.196 X_2_ - 4.6173 X_3_ -11.294 X_1_X_2_ + 2.865 X_1_X_3_ - 9.120 X_2_X_3_ -1.104 X_1_X_2_X_3_ |
| **Weight loss** | WL = 0.517 X_1_ + 2.225 X_2_ - 3.518 X_3_ -0.23394 X_1_X_2_ + 1.580 X_1_X_3_ + 7.807 X_2_X_3_ -2.818 X_1_X_2_X_3_ |
| **Lightness** | L = 16.663 X_1_ - 23.666 X_2_ + 26.340 X_3_ + 8.783 X_1_X_2_ - 4.111 X_1_X_3_ - 22.372 X_2_X_3_ + 10.817 X_1_X_2_X_3_ |
| **Anthracnose incidence** | IA = 3.244 X_1_ - 48.87 X_2_ + 8.689 X_3_ + 16.26 X_1_X_2_ - 0.699 X_1_X_3_ + 111.613 X_2_X_3_ - 35.883 X_1_X_2_X_3_ |

| **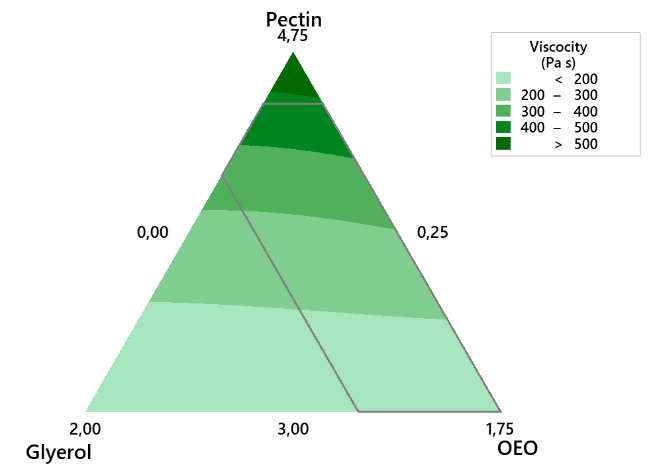a** | **b**  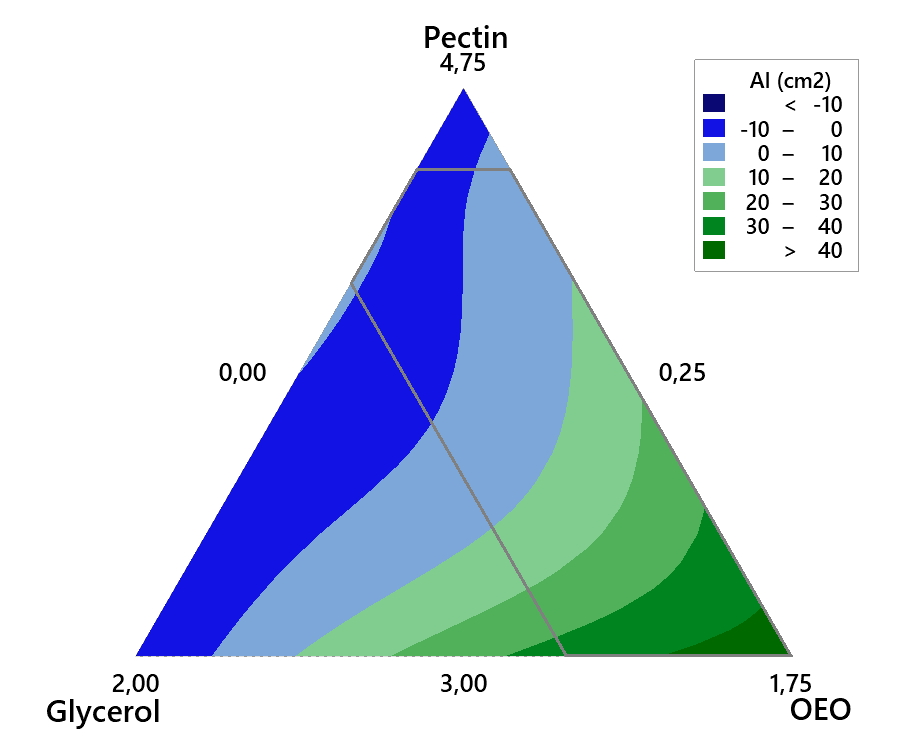 |
| --- | --- |

**Fig. S1.** Contour plots of coating solutions. a) Viscosity and b) IAA. The gray lines within the triangle are the constrained experimental region.


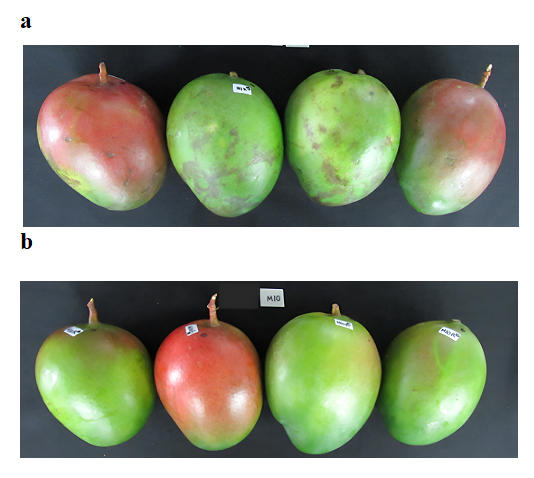


**Fig. S2**. Mango coated with a) Coating solution 1 (3% Pectin, 0.85% Glycerol, 1.15% OEO) and b) Coating solution 10 (3.58% Pectin, 0.85% Glycerol, 0.13% OEO) after 12 days of storage.

| **a**  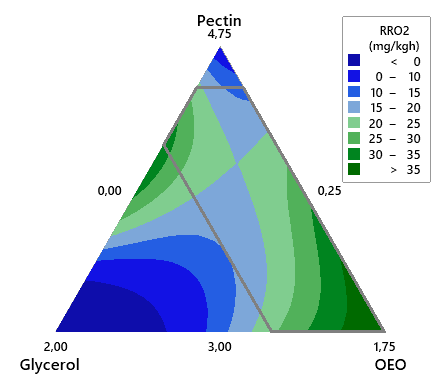 | **b**  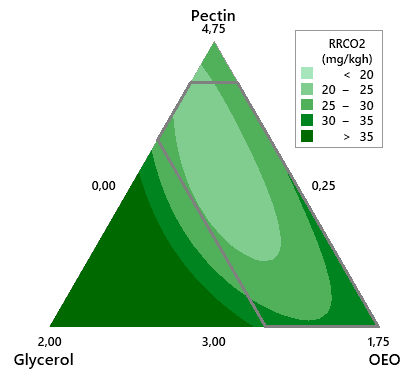 |
| --- | --- |

**Fig. S3**. Contour plots of coated mango respiration rate. a) RRO_2_ and b) RRCO_2_. The gray lines within the triangle are the constrained experimental region.

| **A**  **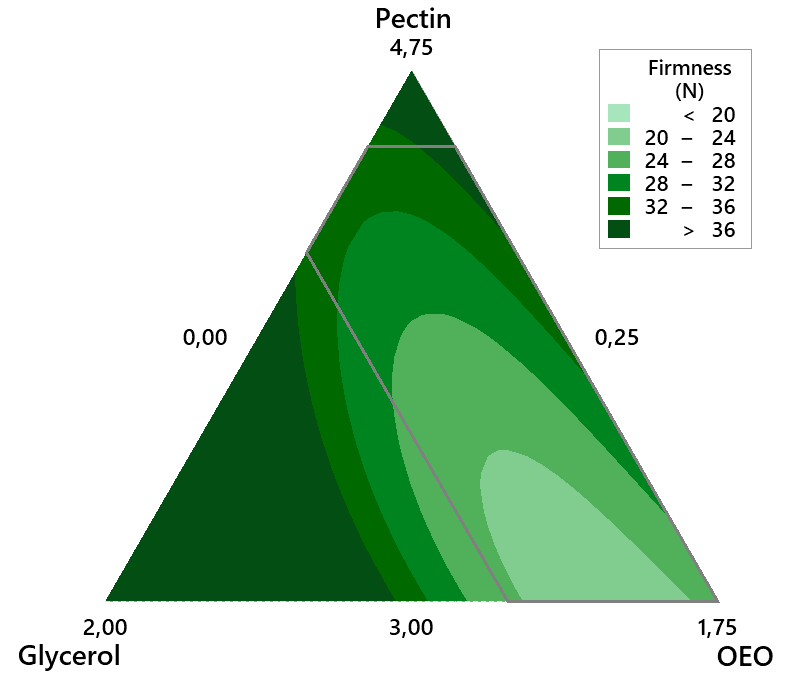** | **B**  **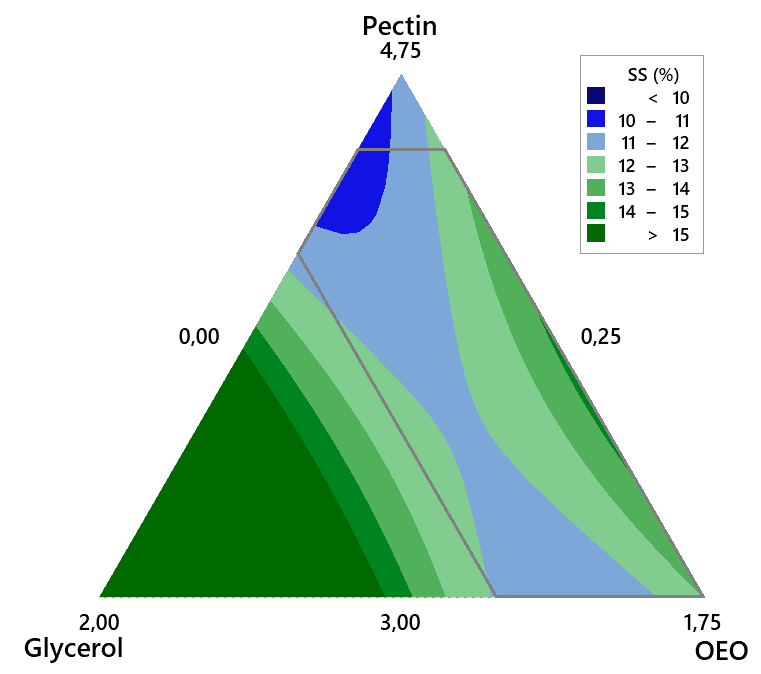** |
| --- | --- |
| **C**  **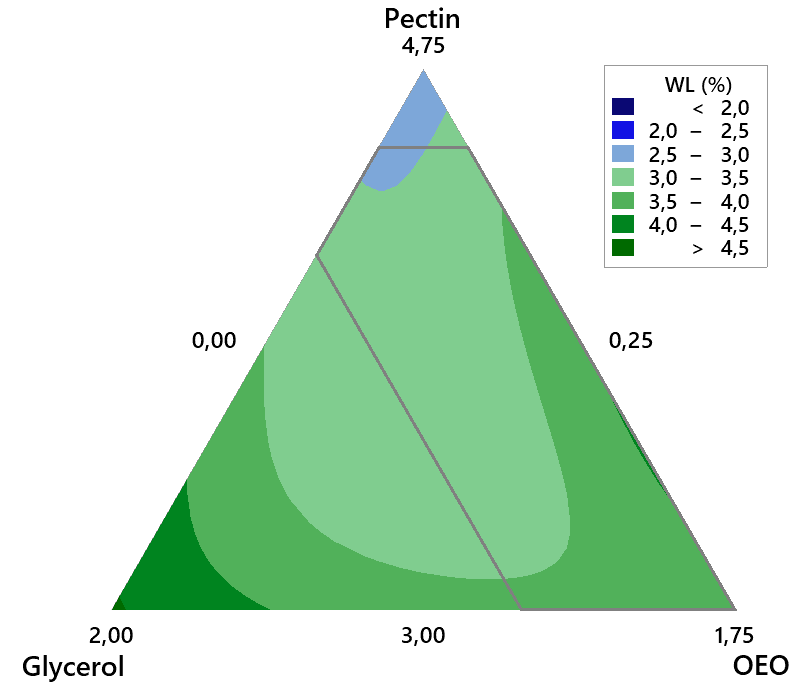** | **D**  **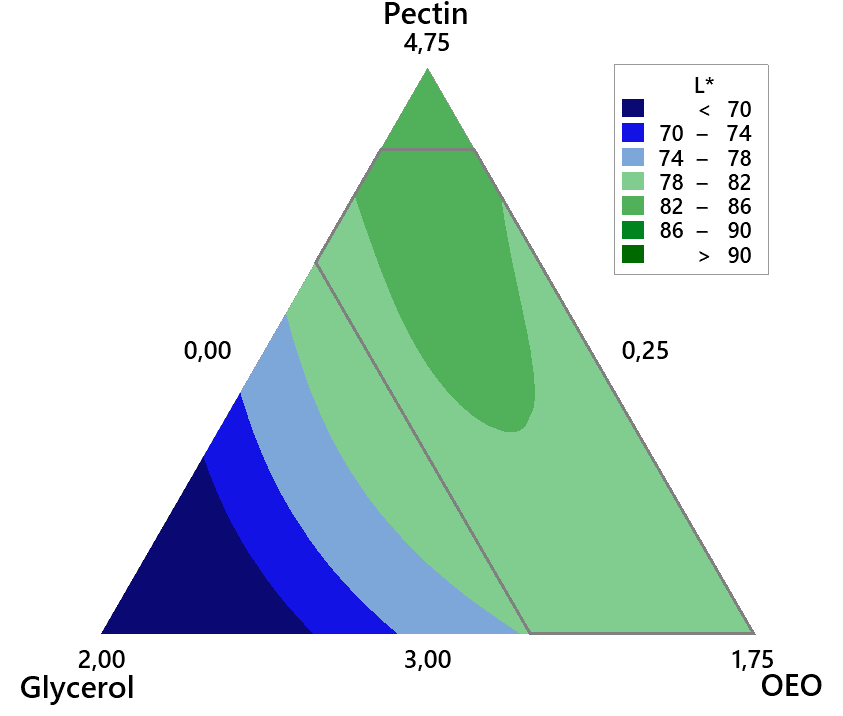** |
| **E**  **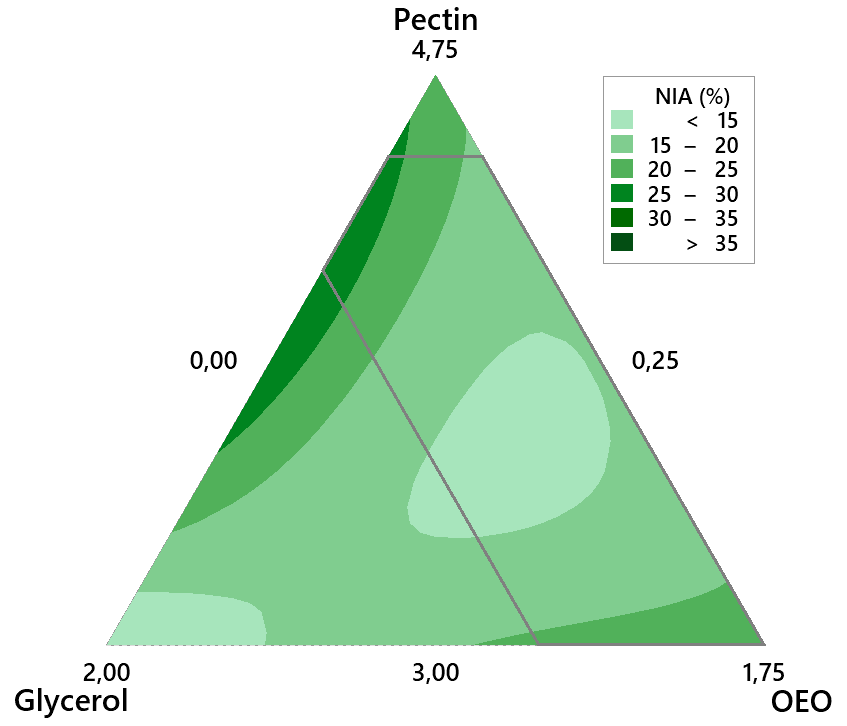** | |

**Fig. S4**. Contour plots of coated mango quality parameters. a) Firmness, b) Soluble solids (SS), c) Weight loss, d) Brightness (L*), and e) Anthracnose incidence (NIA). The gray lines within the triangle are the constrained experimental region.

| **a**  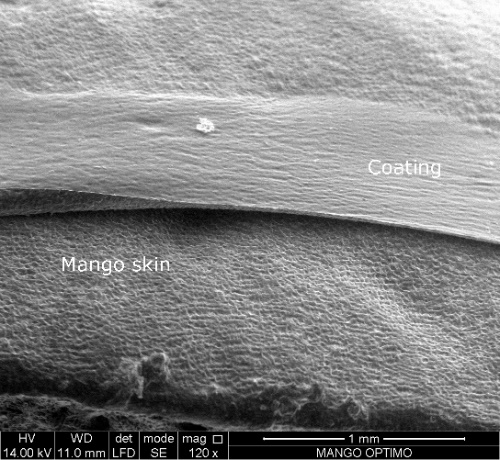 | **b**  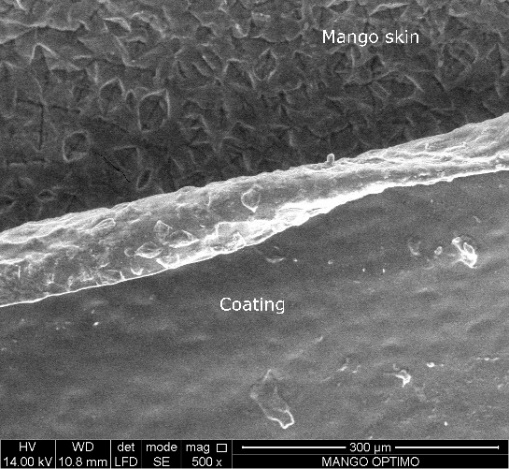 |
| --- | --- |

**Fig. S5.** ESEM micrographs of mango coated with optimal formulation. a) Surface of mango skin. 120X magnification. b) Surface of mango skin. 500 X magnification.

| **a**  **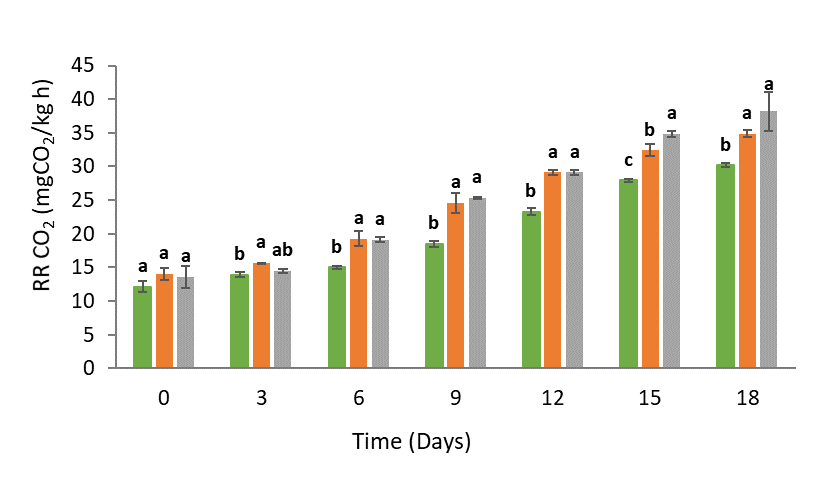** | **b**  **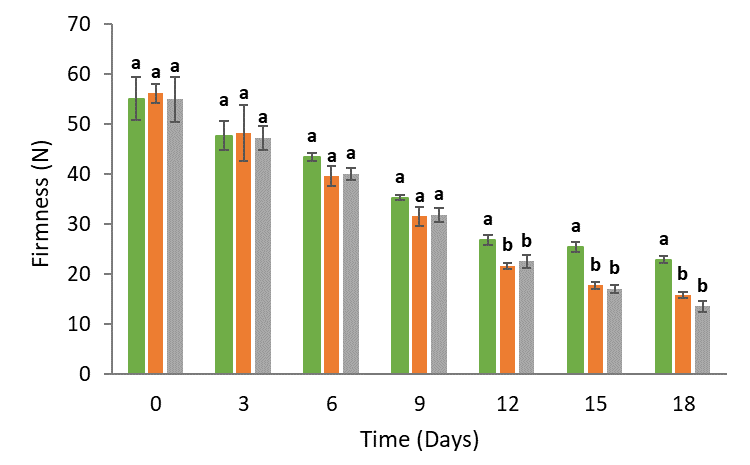** |
| --- | --- |
| **c**  **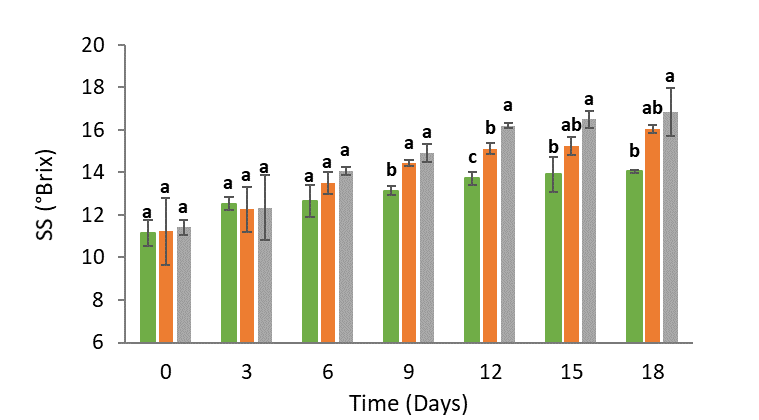** | **d**  **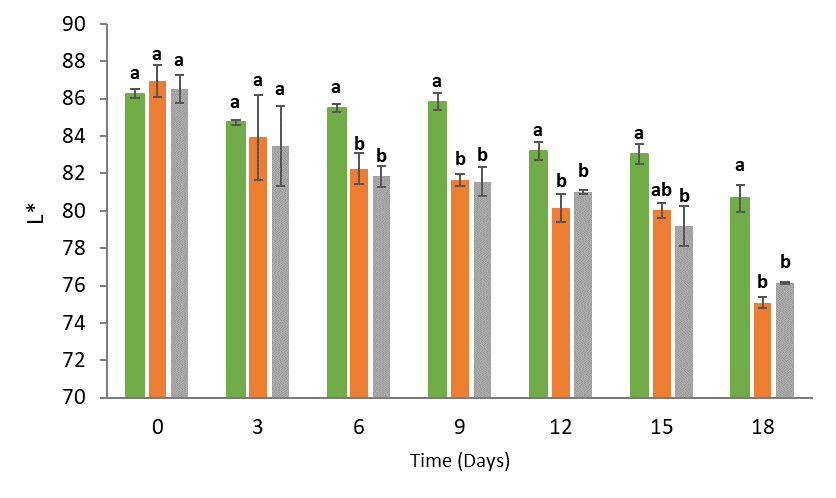** |
| **e**  **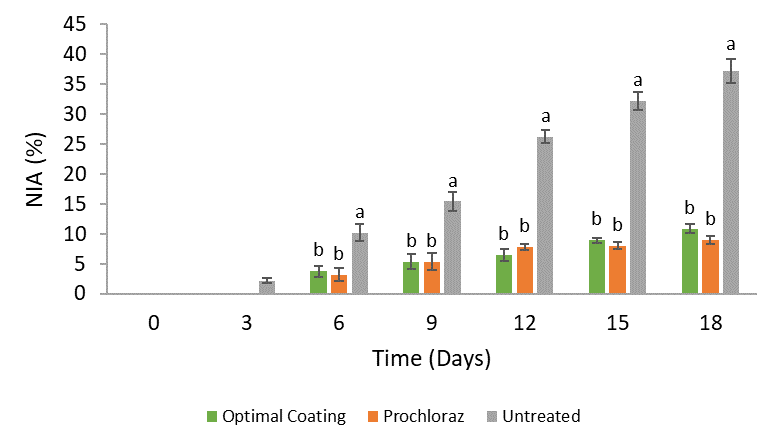** | |

**Fig. S6**. Mango quality parameters treated with the optimal coating (green bars), fungicide Prochloraz (orange bars), and untreated (grey bars). a) RRCO_2_, b) Firmness, c) SS, d) Lightness, and d) NIA
